# Physiological Chemistry

**Published:** 1900-03

**Authors:** John A. Miller

**Affiliations:** Buffalo, N.Y.


### Progress in Medical Science.

### PHYSIOLOGICAL CHEMISTRY.

By JOHN A. MILLER, Ph. D., Buffalo, N. Y.

### TOXALBUMIN FROM THE COMMON EEL.

Elophe Bénech (*Compt. Rend.*, 1899; *J. Chem. Soc.*, 1899) The flesh of the common eel contains a toxalbumin, which, when injected into the vein of the ear of a rabbit, in the proportion of 0.02 gram per kilogram of body weight, causes paralysis, followed by death in about six hours, the blood being dark colored and partially coagulated. It is precipitated by ammonium sulphate, but not by magnesium sulphate. Acetic, hydrochloric, sulphuric, and nitric acids produce a slight turbidity, soluble in excess, but alkalies have no action; alcohol produces an abundant coagulum. The substance gives the biuret reaction, and reactions with tannin, platinic chloride, and the reagents of Tauret, Esbach, Mehn and Millon. When dried it is a yellowish-white powder with a disagreeable taste and an acid after-taste.

---

### ACTION OF IODINE AND IODINE COMPOUNDS.

R. Heinz (*Virchow's Archiv.*, 1899; *J. Chem. Soc.*, 1899). Iodine injected into the serous cavities sets up an adhesive form of inflammation, necrosis of the tissues and increased diapedesis of the blood corpuscles. Of the chloride, bromide and iodide of sodium, the last named is the most powerful. The chlorate, bromate and iodate act as oxidising agents, but only the first-named produces methalmoglobin. Special attention was paid to the activity of sodium iodide; it produces an increase in the leucocytes and of lymph formation. Binz's theory that the action is mainly due to the liberation of iodine is accepted.

---

### ADMINISTRATION OF SUPRARENAL EXTRACT BY THE MOUTH IN HEALTH AND DISEASE.

O. F. F. Gruenbaum (*Proc. Physiol. Soc.*, 1899; *J. Chem. Soc.*, 1899). In health, administration of suprarenal tabloids by the mouth produces no rise of blood pressure, because the extract is not absorbed quickly enough; this renders a good hemostatic, especially in cases of hemorrhage from the walls of the alimentary canal and bladder.

TOXIC ACTION OF SUGARS.

JULIUS VON KOSSA (*Pflüger's Archiv.*, 1899; *J. Chem. Soc.*, 1899). Large doses of sugar injected subcutaneously, or small doses repeated for a long time, produce pathological changes in the organism. The sugars used in the research were principally cane sugar and dextrose; the animals were birds and mammals; the symptoms observed were cyanosis of the comb (in birds), bronchial catarrh, edema of lungs, diarrhea, muscular weakness and sleepiness, incoördination, polydipsia, a gouty state, nephritis and an increased secretion of total nitrogen, urea and ammonia in the urine. Special attention is directed to symptoms similar to those seen in diabetes.

THE FIRST PRODUCT OF THE GASTRIC DIGESTION OF CASEIN.

ERNST SALKOWSKI (*Zeit. Physiol. Chem.*, 1899; *J. Chem. Soc.*, 1899). Under favorable circumstances casein is wholly digested by gastric juice; under unfavorable circumstances (too low a temperature, or too little digestive fluid) there is a residue of paranuclein. Before this, however, there is a primary stage in whch the casein is converted into a proteose which contains all the phosphorus of the original proteid. Casein and caseinogen are in this respect alike.

CHEMISTRY OF MALIGNANT TUMORS.

EUGEN PETRY (*Zeit. Physiol. Chem.*, 1899; *J. Chem. Soc.*, 1899). Extracts of cancerous tumors were made with 0.6 per cent. solution of sodium chloride, then with cold water and finally with dilute alkali. From these extracts, the total proteid was estimated; albumin, globulin and nucleo-proteid were found. There is increase of nucleo-proteid as compared with normal tissues. In a sarcoma, there was very little nucleo-proteid. There is, in these tumors, a considerable amount of proteid which is not coagulable by heat. By autodigestion at the room temperature, this albumose-like proteid increases in quantity.

INFLUENCE OF ALCOHOL ON RESPIRATION IN MAN.

HERMANN WENDELSTADT (*Pflüger's Archiv.*, 1899; *J. Chem. Soc.*, 1899). Large doses of alcohol increase the respiration in most cases in men who are not fatigued, although sometimes only to a small extent. In fatigued individuals, the increase is always seen and is much greater. This action is seen at its best if the alcohol is administered as a wine with rich bouquet.

### TOXIC ACTION OF SODIUM FLUORIDE.

HERBERT B. BALDWIN (*J. Amer. Chem. Soc.*, 1899). Sodium fluoride, taken in doses varying from 0.25 to 9 grams, produces nausea, vomiting and salivation, frequently diarrhea, the effects varying in intensity with the constitution of the individual. In one case, where 10 grams were accidently taken by a man, death ensued within twenty-four hours. In cases of suspected poisoning, the urine may be advantageously tested for fluorine.

---

### PHYSIOLOGICAL SIGNIFICANCE OF ALCOHOL IN THE VEGETABLE KINGDOM.

P. MAZÉ (*Compt. Rend.*, 1899; *J. Chem. Soc.*, 1899). When peas are placed in contact with water, they gradually diminish in weight and in the water relatively large quantities of alcohol are found. Thus 100 peas in 100 c.c. of water at 22-23° yielded 4.63 per cent. of their weight of alcohol in four days and 10.54 per cent. in thirteen days; starch and small quantities of reducing sugars were also found in the water. Peas from which the embryos have been removed give a similar production of alcohol and also a distinct odor of aldehydes. Alcohol, thus appears to be a normal necessary product of the digestion of the carbohydrates of peas during development and it is probably formed at the expense of the glucoses in the living cells by a diastatic process.

---

### PENTOSES IN THE URINE.

ERNST SALKOWSKI (*Zeit. Physiol. Chem.*, 1899; *J. Chem. Soc.*, 1889). The reactions examined for the detection of pentose in the urine were: (1) The Tollens reaction with phloroglucinol and hydrochloric acid; (2) The Tollens reaction with arcinal and hydrochloric acid; (3) the test with aniline acetate paper; (4) the occurrence of furfuraldehyde after distillation with hydrochloric acid; and (5) the preparation of the osazone. Each test is described with full details and the precautions that are necessary. After the use of certain drugs, such as menthol and chloral, these tests, especially the first, may be positive without the presence of pentose; glycuronic acid also may be a source of error. In the presence of dextrose, pentose can still be detected, after the removal of the sugar by fermentation. Three cases of pentosuria are described. The origin of pentose in the body is discussed, and the view that it originates from the nucleoproteids in the pancreas is dismissed. It is regarded as probable that it originates from hexoses.

DETECTION OF ALBUMEN IN URINE.

GABRIEL GUÉRM (*J. Pharm.*, 1899: *J. Chem. Soc.*, 1899). A 10 per cent. aqueous solution of di-iodopara-phenol sulphonic acid (sozoiodol) is a very delicate reagent for the detection of albumin in urine. 10-15 drops of the reagent when added to 8 or 10 c.c. of the filtered urine produce a whiteish, flocculent precipitate or a milky turbidity if albumin is present. Albumoses, peptones and some alkaloids are also precipitated by it, but their precipitates readily dissolve on heating, whereas that produced by albumin is completely insoluble. Alkaline urates and uric acid are precipitated by this reagent.

